# Post-Severe-COVID-19 Cardiopulmonary Rehabilitation: A Comprehensive Study on Patient Features and Recovery Dynamics in Correlation with Workout Intensity

**DOI:** 10.3390/jcm12134390

**Published:** 2023-06-29

**Authors:** Andreea Dumitrescu, Gabriela Doros, Voichita Elena Lazureanu, Susa Septimiu-Radu, Felix Bratosin, Ovidiu Rosca, Harshkumar Patel, Tamara Mirela Porosnicu, Gabriela Mut Vitcu, Andrei Mirea, Cristian Oancea, Stefan Mihaicuta, Emil Robert Stoicescu, Paula Irina Barata

**Affiliations:** 1 Cardioprevent Foundation, Calea Dorobantilor 3, 300134 Timisoara, Romania; andreea.dumitrescu@cardioprevent.org (A.D.); gabriela.mut@cardioprevent.org (G.M.V.); andrei.mirea98@yahoo.ro (A.M.); stefan.mihaicuta@umft.ro (S.M.); 2 Third Discipline of Pediatrics, “Victor Babes” University of Medicine and Pharmacy Timisoara, Eftimie Murgu Square 2, 300041 Timisoara, Romania; gdoros@gmail.com; 3 Department XIII, Discipline of Infectious Diseases, “Victor Babes” University of Medicine and Pharmacy Timisoara, Eftimie Murgu Square 2, 300041 Timisoara, Romania; lazureanu.voichita@umft.ro (V.E.L.); felix.bratosin@umft.ro (F.B.); ovidiu.rosca@umft.ro (O.R.); 4 Doctoral School, “Victor Babes” University of Medicine and Pharmacy Timisoara, Eftimie Murgu Square 2, 300041 Timisoara, Romania; mirela.porosnicu@umft.ro; 5 Department of General Medicine, Pandit Deendayal Upadhyay Medical College, Rajkot 360001, Gujarat, India; harshpatel311133@gmail.com; 6 Intensive Care Unit, “Victor Babes” Clinical Hospital for Infectious Diseases and Pneumology, 300041 Timisoara, Romania; 7 Center for Research and Innovation in Precision Medicine of Respiratory Diseases, “Victor Babes” University of Medicine and Pharmacy Timisoara, Eftimie Murgu Square 2, 300041 Timisoara, Romania; oancea@umft.ro; 8 Discipline of Physiology, “Victor Babes” University of Medicine and Pharmacy Timisoara, Eftimie Murgu Square 2, 300041 Timisoara, Romania; stoicescu.emil@umft.ro (E.R.S.); barata.paula@student.uvvg.ro (P.I.B.); 9 Department of Physiology, Faculty of Medicine, “Vasile Goldis” Western University of Arad, Revolutiei Square 94, 310025 Arad, Romania

**Keywords:** COVID-19, SARS-CoV-2, post acute COVID-19 syndrome, cardiopulmonary rehabilitation

## Abstract

The aftermath of severe COVID-19 frequently involves considerable cardiopulmonary damage, necessitating rehabilitation. This study aimed to evaluate the impact of COVID-19 on cardiopulmonary health and assess the effectiveness of various rehabilitative interventions. Conducted between September 2021 and September 2022, this prospective study included patients who had been diagnosed with severe COVID-19 and admitted at the “Victor Babes” Infectious Diseases and Pulmonology Hospital, Timisoara, Romania. The patients were stratified into low- and high-intensity rehabilitation groups. The rehabilitation protocols were individually tailored, and the patient recovery was closely monitored over a 3-month period. Our cohort comprised 84 patients, with a mean age of 56.3 years for the low-intensity group (*n* = 42) and 53.1 years for the high-intensity group (*n* = 42). Both groups showed significant improvements in the lung injury area, need for oxygen supplementation, ejection fraction, systolic pulmonary artery pressure, and forced vital capacity. Additionally, considerable enhancements were observed in maximal voluntary ventilation, FEV1, FEV1/FVC ratio, peak expiratory flow, and forced expiratory flow at 25–75%. The work intensity also demonstrated substantial improvements from the initial testing to the 3-month mark in both groups. This study provides evidence that personalized, targeted rehabilitation strategies can improve long-term cardiopulmonary health in patients recovering from severe COVID-19, proving both low-intensity and high-intensity training as sufficient to improve heart and lung function if performed correctly and over a relatively short duration of 3 months. The study findings underscore the importance of implementing comprehensive cardiopulmonary rehabilitation protocols in the care of post-COVID-19 patients and highlight the value of stratified rehabilitation intensity based on individual patient dynamics and recovery features.

## 1. Introduction

The global COVID-19 pandemic has proven to be a significant health challenge, with long-term implications extending far beyond the acute phase of infection [1]. One critical area of growing concern involves the aftermath of COVID-19 infection on cardiopulmonary function, as this disease has highlighted both acute and chronic implications for these vital systems [2,3,4]. Patients with severe COVID-19 often present with respiratory distress and other cardiopulmonary complications, with many patients experiencing continued issues even after recovery from the acute phase of the virus [5,6]. The prolonged and, in some cases, enduring effects on cardiopulmonary health post-COVID-19 have initiated the need for targeted rehabilitation approaches [7].

These so-called “long-COVID” or post-acute sequelae of SARS-CoV-2 infection have underscored the importance of having a comprehensive understanding of the varied and multifaceted impact of the virus [8]. Cardiopulmonary complications can severely impact the quality of life, making it essential to optimize recovery and facilitate a return to routine activities. In particular, there is a pressing need to understand the mechanisms behind these sequelae and develop an effective rehabilitation strategy, leading to individualized treatment plans based on patient features and recovery dynamics [9,10].

Numerous studies have documented the cardiopulmonary consequences of COVID-19 [11,12,13]. However, there is a relative shortage of research focusing on effective rehabilitation strategies. These rehabilitative interventions can play a critical role in the ameliorating physical function, exercise capacity, and health-related quality of life in these patients [14]. An in-depth understanding of the physiological and psychological impact of the disease will inform these strategies, encouraging comprehensive, tailored recovery plans [15,16].

Considering the broad range of individual patient experiences and the diverse course of recovery, it is crucial to implement a personalized approach to rehabilitation. In this context, the patient’s demographic features, clinical characteristics, and recovery dynamics can provide valuable insights into tailoring effective rehabilitation programs. Such a personalized approach has the potential to expedite recovery, minimize residual symptoms, and significantly improve the quality of life for patients recovering from COVID-19 [17]. Against this backdrop, there is a pressing need for well-designed studies to elucidate the patient-specific factors influencing recovery and to determine the most effective rehabilitation strategies [18]. 

Therefore, the primary objective of this comprehensive study is to evaluate the impact of COVID-19 on cardiopulmonary health and assess the effectiveness of various rehabilitative interventions. This study hypothesizes that post-COVID-19 patients display distinctive features and recovery dynamics, which significantly influence their response to specific rehabilitative interventions. We aim to identify these features and dynamics based on different training plans and intensities in order to develop personalized, targeted rehabilitation strategies that can enhance recovery and improve long-term cardiopulmonary health among patients that have recovered from severe COVID-19.

## 2. Materials and Methods

### 2.1. Study Design and Ethics

This study was a prospective analysis conducted for one year, between September 2021 and September 2022, during the COVID-19 pandemic. The patients included in the study were admitted with severe COVID-19 at the Infectious Diseases and Pulmonology Hospital “Victor Babes”, which is affiliated with the “Victor Babes” University of Medicine and Pharmacy from Timisoara, Romania. Ethical approval for the study was obtained from the Institutional Review Board of the hospital. All the patient data were anonymized before analysis to maintain confidentiality.

### 2.2. Patients’ Inclusion and Exclusion Criteria

A convenience sampling method was employed to estimate the appropriate size of the patient sample, which was calculated for 35 patients for each patient group, at a confidence level of 95% and a margin of error of 10%. The patients included in the study were adults (18 years or older) who had been diagnosed with severe COVID-19, as defined by the World Health Organization [19]. The diagnosis had been confirmed via a positive SARS-CoV-2 PCR test [20], and the medical histories of the patients were retrieved from the paper and digital records based on the ICD-10 diagnostic codes [21]. All the records were checked for the patient’s consent to participate in medical research studies. The patients were proportionally matched by age, gender, and vaccination status to reduce bias. Further stratification was implemented based on the type of rehabilitation protocol (low intensity or high intensity) by randomization, resulting in two equal study groups that underwent low-intensity and high-intensity workout, respectively. The rehabilitation program started at discharge from the infectious diseases department, after the SARS-CoV-2 infection was cleared and confirmed via a PCR test. 

**Figure 1:**
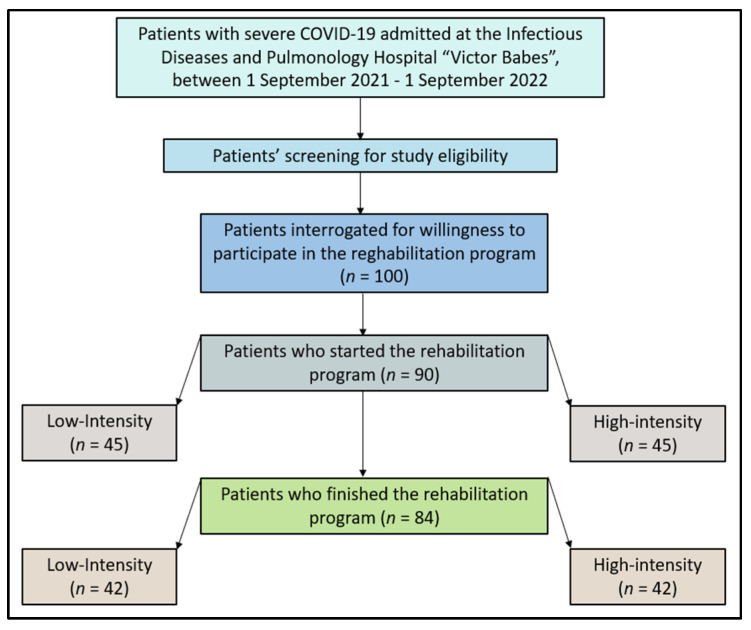
Study flowchart.

Patients were excluded from the study if they had a known history of immunodeficiency diseases, were receiving immunosuppressive therapy before the onset of COVID-19, or if they had a history of chronic lung disease. To avoid confounding factors, patients with severe COVID-19 that required ICU admission during hospitalization were also not considered for this study due to a higher prevalence of functional impairment and pulmonary structural abnormalities. Pregnant women and patients who did not provide consent for their medical data to be used for research purposes were excluded from the study. Additionally, patients with severe and unstable cardiovascular conditions such as recent myocardial infarction, unstable angina, uncontrolled arrhythmias, or decompensated heart failure may be contraindicated from participating in the study. Patients with severe and uncontrolled respiratory conditions, such as acute exacerbation of chronic obstructive pulmonary disease (COPD), severe asthma, or pulmonary hypertension, may be contraindicated from enrolling. Patients who developed active infectious diseases, pneumonia, or other acute respiratory infections were contraindicated to continue participating in the study. Lastly, patients with severe cognitive impairments or mental health conditions that affect their ability to understand and follow instructions, participate in therapy sessions, or provide informed consent were contraindicated to enroll. A total of three patients from each group, who did not complete the rehabilitation program, were not included in the final analysis, as presented in Figure 1.

The patient categorization into the severe COVID-19 group was based on specific clinical and paraclinical features, aligning with the World Health Organization’s definition and various clinical guidelines [19]. Severe COVID-19 was diagnosed if patients exhibited any of the following clinical features: dyspnea and a respiratory rate of 30 breaths per minute or more in resting conditions. An oxygen saturation (SpO2) of less than 93% of room air at sea level was a critical indicator. Alternatively, patients requiring supplemental oxygen to maintain an SpO2 above this threshold were also considered severe. Clinical signs of pneumonia with severe cough, fever, and other signs suggestive of lower respiratory tract infection were also taken into account.

Alongside the clinical symptoms, paraclinical features were also considered to provide a more comprehensive understanding of the severity of COVID-19 in the patients. Radiological findings that were consistent with severe pneumonia, such as extensive bilateral infiltrates on chest X-ray or high-resolution computed tomography (HRCT) of the chest, were considered. The laboratory parameters included elevated D-dimer levels, lymphopenia (low lymphocyte count), elevated C-reactive protein (CRP), elevated liver enzymes (TGO, TGP), and increased levels of inflammatory markers like IL-6 and ferritin. Evidence of respiratory compromise, such as a partial pressure of oxygen (PaO2) to the fraction of inspired oxygen (FiO2) ratio of less than 300 or the presence of acute respiratory distress syndrome (ARDS) as per the Berlin definition [22], were also considered. 

### 2.3. Rehabilitation Protocols

The cardiopulmonary rehabilitation protocols, given the age-specific needs and potential comorbidities of our study participants, were diligently tailored to ensure they were age-appropriate, safe, and effective.

The low-intensity protocol involved initial training on a cyclo-ergometer, starting with an effort load of 20–30 Watts, which is equivalent to approximately 40–50% of the patient’s estimated maximum effort. The training sessions were scheduled for three times a week. As the patient’s endurance improved, gradual walking exercises were incorporated, initially indoors, progressing to outdoors. Breathing exercises were included, emphasizing guided coughing exercises to clear airways and drainage postures to aid in the removal of excess mucus. Strength training, involving low-weight resistance exercises using 1–2 kg dumbbells or low-elasticity resistance bands, was incorporated twice a week. Flexibility exercises were conducted 2–3 times a week. Educational sessions were integral to the protocol, focusing on teaching the patient to gauge their level of exertion and recognize signs of over-exertion. Each exercise session, including the warm-up, main exercise, and cool-down, was approximately 45–60 min in duration.

The high-intensity protocol consisted of more rigorous training. The aerobic exercises on the cyclo-ergometer were increased to an effort load of 40–50 Watts, roughly 60–70% of the patient’s estimated maximum effort, with the frequency increased to five times a week. For the patients showing a favorable response, interval training, with alternating periods of increased and reduced effort, was introduced. Breathing exercises were supplemented with inspiratory muscle training using a threshold device to enhance respiratory muscle strength and endurance. The resistance in the strength training was gradually increased, and the frequency of training was set at 3–4 times a week. For patients demonstrating good progress, high-intensity interval training (HIIT) was introduced cautiously, with careful monitoring of the patient’s tolerance and response. Educational sessions were extended to cover additional topics such as stress management, coping strategies, and nutrition advice. Each exercise session under the high-intensity protocol, including rest periods between different exercises, lasted approximately 60–90 min.

Regardless of the intensity of the protocol, kinesiotherapy was a common element aimed at improving body mechanics, balance, and coordination. This was intended to benefit patients who had experienced any degree of functional decline due to illness or prolonged hospitalization. The rehabilitation team guided patients through a range of therapeutic exercises designed to restore strength, mobility, and function while also mitigating the risk of falls and injuries.

### 2.4. Study Variables

The clinical data were retrieved from the patients’ paper and electronic medical records. We collected a wide array of data points to achieve a holistic understanding of the patients’ clinical status, recovery dynamics, and rehabilitation outcomes. The variables included demographic information, laboratory data, pulmonary function measures, cardiopulmonary exercise testing parameters, and recovery protocol data:

(1) Demographic and lifestyle variables: This included age, gender, vaccination status, pre-COVID-19 level of physical activity, Body Mass Index (BMI), duration of hospitalization, and patient comorbidities;

(2) Laboratory data: A series of blood tests were carried out to measure the following variables: leukocyte count, lymphocyte count, hemoglobin (Hb), aspartate transaminase (AST, also known as TGO), alanine transaminase (ALT, also known as TGP), creatinine, C-reactive protein (CRP), interleukin-6 (IL-6), procalcitonin, D-dimers, and ferritin. These were aimed at assessing the general health, inflammatory status, and coagulation profile of the patients;

(3) Pulmonary and cardiac measures: These included measures such as lung injury percentage, oxygen requirement (O2 need), the ejection fraction (EF), systolic pulmonary artery pressure (PSAP), and lung function measures such as maximal voluntary ventilation (MVV), forced vital capacity (FVC), forced expiratory volume in 1 s (FEV1), FEV1/FVC ratio, peak expiratory flow (PEF), and forced expiratory flow at 25–75% (FEF 25–75);

(4) Cardiopulmonary exercise testing variables: These comprised work (W), work from predicted (W from predicted), oxygen consumption at peak exercise (VO2 peak), VO2 peak from predicted, oxygen consumption at anaerobic threshold (VO2 at AT), the anaerobic threshold from maximum (AT from max), respiratory exchange ratio (RER), maximum heart rate (FC from max), delta heart rate per delta VO2 (delta HR per delta VO2), delta VO2 per delta work (delta VO2 per delta W), oxygen consumption per heart rate (VO2 per HR), and the breathing reserve (BR);

(5) Antiviral medication requirement: We recorded the need for antiviral medication during the acute phase of the disease, which provides insights into the severity of the initial infection;

(6) Cardiopulmonary rehabilitation protocol: We recorded the type of rehabilitation protocol (low or high intensity) assigned to each patient. The above variables were collected at the beginning (indicated with an ‘i’) and end of the rehabilitation period (indicated with an ‘f’) to compare baseline values and post-rehabilitation outcomes, respectively.

### 2.5. Statistical Analysis

The statistical analyses were performed using SPSS software (version 26.0). Descriptive statistics were used to characterize the study population, presenting continuous variables as means and standard deviations and categorical variables as frequencies and percentages. For the comparison of the proportions, the Chi-square and Fisher’s tests were employed, while, for the comparison of group differences in the nonparametric data, the Mann–Whitney test was used. The parametric continuous variables that followed a normal distribution were compared via a mean and standard deviation using the Student’s *t*-test (unpaired, independent samples). The median and IQR values for the nonparametric data were compared using the Mann–Whitney u-test. All the tests were two-sided, and *p*-values less than 0.05 were considered statistically significant.

## 3. Results

### 3.1. Patients’ Background

In the demographic and lifestyle variables presented in Table 1, the age distribution in both groups did not differ significantly, with a mean age of 56.3 years for the low-intensity group and 53.1 years for the high-intensity group. The sex distribution across both groups also showed no significant difference, with men comprising 54.8% (*n* = 23) and 59.5% (*n* = 25) in the low- and high-intensity groups, respectively, while women comprised 45.2% (*n* = 19) and 40.5% (*n* = 17) of the low- and high-intensity groups, respectively. In the low-intensity group, 19.0% of the patients were obese, compared with 14.3% in the high-intensity group (*p* = 0.669). The COVID-19 vaccination status also did not significantly differ between the groups (*p* = 0.629), with 26.2% (*n* = 11) and 31.0% (*n* = 13) of the patients in the low- and high-intensity groups, respectively, having received two doses of the vaccine. The unvaccinated patients constituted 73.8% (*n* = 31) of the low-intensity group and 69.0% (*n* = 29) of the high-intensity group. The level of physical activity showed no significant difference (*p* = 0.512) across the two groups. In the low-intensity group, 61.9% (*n* = 26) were sedentary, 28.6% (*n* = 12) were active, and 9.5% (*n* = 4) were very active. The high-intensity group had 52.4% (*n* = 22) sedentary, 40.5% (*n* = 17) active, and 7.1% (*n* = 3) very active

### 3.2. Laboratory Data

Table 2 summarizes the laboratory data measured after SARS-CoV-2 viral clearance for both the low- and high-intensity groups. Regarding white blood cell (WBC) counts, the difference between the low-intensity group (median 11.4) and the high-intensity group (median 10.6) was not statistically significant (*p* = 0.649). A similar trend was observed for the lymphocyte counts, where the median values were 6.1 and 6.4 for the low- and high-intensity groups, respectively (*p* = 0.345). Additionally, no significant difference was found between the two groups for hemoglobin levels, liver function tests, and creatinine levels.

Regarding the inflammatory markers, the C-reactive protein (CRP) and Interleukin-6 (IL-6) levels also did not significantly differ between the two groups (*p* = 0.190 and *p* = 0.662, respectively). The median CRP levels were 24 (IQR 16) in the low-intensity group and 28 (IQR 14) in the high-intensity group, while the median IL-6 levels were 9.8 (IQR 4.7) and 12.9 (IQR 5.8), respectively. The difference in the procalcitonin values was not statistically significant between the groups (*p* = 0.457), with median values of 0.10 (IQR 0.04) in the low-intensity group and 0.12 (IQR 0.06) in the high-intensity group. Similarly, the D-dimers showed no significant difference (*p* = 0.619), with median values of 231 (IQR 96) in the low-intensity group and 244 (IQR 101) in the high-intensity group. Finally, ferritin did not significantly differ between the two groups (*p* = 0.334), with median values of 206 (IQR 48) in the low-intensity group and 225 (IQR 62) in the high-intensity group.

### 3.3. Cardiopulmonary Measurements

Table 3 presents the initial pulmonary measurements at discharge, where the extent of lung injury was not significantly different between the low-intensity group, with a median of 21%, and the high-intensity group, with a median of 23% lung injury. Similarly, the proportion of the patients requiring oxygen supplementation showed no significant difference between the two study groups. In terms of the systolic pulmonary artery pressure (SPAP), the two groups showed no significant difference (*p* = 0.713), with median values of 30 mmHg (IQR 28–32) and 29 mmHg (IQR 27–31) for the low- and high-intensity groups, respectively. The maximal voluntary ventilation (MVV) was also similar in both groups (*p* = 0.699), with a median of 75 L/min (IQR 71–82) in the low-intensity group and 78 L/min (IQR 73–83) in the high-intensity group. No significant difference was found between the two groups for a forced vital capacity (FVC) (*p* = 0.527), with median values of 73% predicted (IQR 70–79) and 76% predicted (IQR 72–81) in the low- and high-intensity groups, respectively. Other variables that showed no significant differences at measurement between the two study groups were the forced expiratory volume in 1 s (FEV1), the FEV1/FVC ratio, the expiratory flow (PEF), and the forced expiratory flow at 25–75% (FEF 25–75), with median values of 78% predicted (IQR 74–82) in the low-intensity group and 81% predicted (IQR 77–85) in the high-intensity group.

Table 4 reports the pulmonary measurements of the patients with severe COVID-19 following three months of rehabilitation. In terms of lung injury, both groups exhibited a decrease compared to the initial measurements post-discharge, where the patients in the low-intensity group showed a median lung injury of 14%, while those from the high-intensity group reported a median of 12% of the affected lung area without significant differences. The oxygen needs also decreased in both groups after three months of rehabilitation. The low-intensity group showed a median oxygen need of 20% (IQR 18–22), and the high-intensity group reported a median of 19% (IQR 17–21). The difference was not statistically significant (*p* = 0.441). The systolic pulmonary artery pressure (SPAP) was also similar for both groups (*p* = 0.210), with median values of 25 mmHg (IQR 23–27) and 24 mmHg (IQR 22–26) for the low- and high-intensity groups, respectively. Similar findings were observed when measuring the maximal voluntary ventilation (MVV), and the forced vital capacity (FVC). 

The forced expiratory volume in 1 s (FEV1) was also similar between the two groups (*p* = 0.708), with the low-intensity group reporting a median of 83% predicted (IQR 82–86) and the high-intensity group reporting a median of 87% predicted (IQR 85–91). There was no significant difference in the FEV1/FVC ratio between the two groups (*p* = 0.662), with median values of 75% (IQR 72–78) in the low-intensity group and 76% (IQR 74–78) in the high-intensity group. Similarly, the peak expiratory flow (PEF) did not significantly differ between the two groups (*p* = 0.317), with median values of 81% predicted (IQR 78–85) in both groups. Lastly, the forced expiratory flow at 25–75% (FEF 25–75) showed no significant difference between the groups (*p* = 0.301), with median values of 84% predicted (IQR 82–90) in the low-intensity group and 83% predicted (IQR 81–87) in the high-intensity group.

### 3.4. Cardiopulmonary Exercise Testing

Table 5 presents the initial cardiopulmonary exercise testing results of the patients with severe COVID-19 at discharge. Regarding work intensity, measured in Watts (W), there was a significant difference (*p* < 0.001) between the low- and high-intensity groups, which exhibited median values of 27 (22–29) and 43 (41–46), respectively. The oxygen consumption at peak exercise (VO2 peak) revealed that 52.4% of the low-intensity group and 57.1% of the high-intensity group fell below the normal range of >20 mL/kg/min. However, the difference was not statistically significant (*p* = 0.294). For the VO2 from predictions (expected VO2 for age, gender, and size), no significant difference was found between the low- and high-intensity groups (*p* = 0.708), with median values being within the normal range for both groups.

In terms of the VO2 at the anaerobic threshold (AT), the median values of the low-intensity group and high-intensity group were 10 mL/kg/min (IQR 9–11) and 11 mL/kg/min (IQR 10–12), respectively, which were both below the normal range (>11 mL/kg/min). However, the difference was not statistically significant (*p* = 0.460). Similarly, for the respiratory exchange ratio (RER) and the change in the heart rate relative to the change in oxygen consumption (ΔHR/ΔVO2), both groups exhibited median values slightly above the normal range but without statistically significant differences. The change in the oxygen consumption relative to work intensity (ΔVO2/W) was below the normal range (10–15 mL/min/W) for both groups, and there was no significant difference (*p* = 0.783) between them. Similarly, the change in oxygen consumption relative to heart rate (ΔVO2/HR) was below the normal range (10–15 mL/beat) for both groups, with no significant difference (*p* = 0.419) between them. Finally, the breathing reserve (BR) was below the normal range (20–40%) for both groups, with median values of 15% (IQR 12–18) and 18% (IQR 15–21) for the low- and high-intensity groups, respectively. The difference was not statistically significant (*p* = 0.301).

Table 6 outlines the final cardiopulmonary exercise testing results of the patients with severe COVID-19 after three months of rehabilitation. The work intensity improved for both groups, with a higher median for the high-intensity group (38W, IQR 36–44) compared to the low-intensity group (47W, IQR 44–51). Despite this, the *p*-value of 0.094 suggested that this difference was not statistically significant. The peak oxygen consumption (VO2 peak) also improved in both groups, rising above the normal threshold of >20 mL/kg/min, but the difference between the groups (*p* = 0.331) was not statistically significant. Similar improvements were observed regarding the VO2 from the predicted value showed and the oxygen consumption at the anaerobic threshold (VO2 at AT). For the respiratory exchange ratio (RER), the median values for both groups fell within the normal range (0.8–1.15), and there was no significant difference (*p* = 0.190) between the two groups. 

The ratio of the change in HR to the change in VO2 (ΔHR/ΔVO2) fell within the normal range (1–1.5) for both groups, with no significant difference (*p* = 0.325) between them. The change in oxygen consumption relative to work intensity (ΔVO2/W) increased for both groups, with no significant difference (*p* = 0.282) between them. Similarly, the change in oxygen consumption relative to heart rate (ΔVO2/HR) also increased for both groups, with no significant difference (*p* = 0.440). The breathing reserve (BR) increased for both groups, with the high-intensity group displaying a higher median (29%, IQR 27–31) compared to the low-intensity group (24%, IQR 22–26). However, the difference between the two groups was not statistically significant (*p* = 0.273).

### 3.5. Final Comparisons

Table 7 offers a comparative view of the initial cardiopulmonary measurements and results after three months between the low-intensity and high-intensity rehabilitation groups. For both low- and high-intensity groups, there was a significant reduction in lung injury percentage from the initial measurement to the 3-month mark. The low-intensity group demonstrated a reduction from a median of 21% to 14% with a *p*-value of 0.005. The high-intensity group showed a similar trend, reducing from 23% to 12% (*p*-value = 0.001). This suggests a significant improvement in lung conditions for patients in both rehabilitation groups. The need for oxygen also saw a notable decrease for both groups. In the low-intensity group, it went down from a median of 30% initially to 20% at the 3-month mark (*p* = 0.002). In the high-intensity group, it decreased from 28% (IQR 23–33) to 19% (IQR 17–21) with a *p*-value of 0.003. These results suggest that the rehabilitation program helped to improve the patient’s oxygenation.

Regarding the fraction of expired oxygen (FE), both groups showed a significant improvement. For the low-intensity group, the median FE increased from 82 to 85 (*p*-value of 0.010). For the high-intensity group, the median increased from 84 to 87 (*p*-value = 0.020). The systolic pulmonary artery pressure (SPAP) decreased significantly in both groups. The low-intensity group saw a reduction from 30 (IQR 28–32) to 25 (IQR 23–27), *p* = 0.015, while the high-intensity group saw a reduction from 29 to 24 (*p* = 0.011). The maximal voluntary ventilation (MVV) increased significantly in both groups, as seen in Figure 1. The low-intensity group saw an increase from 75 (IQR 71–82) to 85 (IQR 82–89), *p*-value <0.001, and the high-intensity group saw an increase from 78 to 87 (*p* = 0.008).

**Figure 2:**
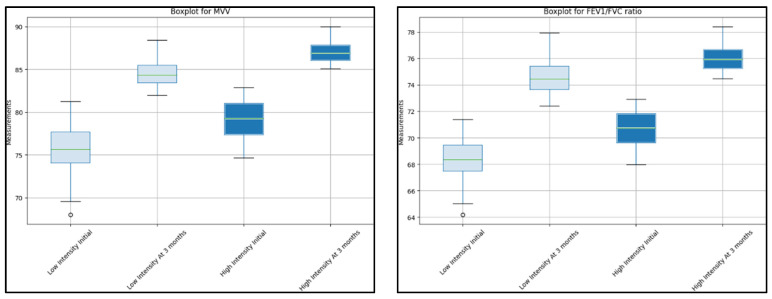
Comparison between initial cardiopulmonary measurements and 3-month results between low-intensity and high-intensity rehabilitation groups.

The forced vital capacity (FVC) increased significantly in both groups. The low-intensity group saw an increase from 73 (IQR 70–79) to 85 (IQR 81–88), *p* = 0.004, while the high-intensity group saw an increase from 76 (IQR 72–81) to 85 (IQR 80–88), *p* = 0.007. The forced expiratory volume in 1 s (FEV1) improved significantly in both groups. The low-intensity group saw an increase from 75 (IQR 70–80) to 83 (IQR 82–86), *p* = 0.009, and the high-intensity group saw an increase from 74 (IQR 70–80) to 87 (IQR 85–91), *p*-value of less than 0.001. The FEV1/FVC ratio improved significantly for both groups, as described in Figure 2. The low-intensity group improved from a median of 68 (IQR 65–71) to 75 (IQR 72–78), *p*-value of less than 0.001, while the high-intensity group improved from 70 (IQR 67–73) to 76 (IQR 74–78), *p* = 0.010. The peak expiratory flow (PEF) showed a significant increase in both groups. The low-intensity group improved from 76 (IQR 72–80) to 81 (IQR 78–85), *p* = 0.012, and the high-intensity group improved from 79 (IQR 75–83) to 81 (IQR 79–85), *p* = 0.020. The forced expiratory flow at 25–75% (FEF 25–75) showed a significant increase for both groups. The low-intensity group improved from a median of 78 (IQR 74–82) to 84 (IQR 82–90), *p* = 0.005, while the high-intensity group improved from 81 to 83 (*p* = 0.022).

Table 8 presents a comparative analysis of the initial and 3-month cardiopulmonary testing results for the low-intensity and high-intensity rehabilitation groups. For work intensity (W), the low-intensity group showed a significant increase from initial testing to the 3-month mark, from a median of 27 (IQR 22–29) to 38 (IQR 36–44), (*p*-value < 0.001), while the high-intensity group increased from 43 (IQR 41–46) to 47 (IQR 44–51), (*p*-value = 0.135). In terms of the oxygen consumption at peak exercise (VO2 (peak)), both groups showed a significant improvement. The low-intensity group improved from 18 (IQR 17–19) to 21 (IQR 20–22), *p* = 0.010, and the high-intensity group improved from 19 (IQR 18–20) to 23 (IQR 22–24), *p* = 0.002.

The VO2 from the predictions demonstrated significant increases for both groups. The low-intensity group improved from 75 (IQR 70–80) to 85 (IQR 80–90), *p* = 0.003, and the high-intensity group improved from 78 (IQR 73–83) to 90 (IQR 85–95), *p*-value of less than 0.001. The oxygen consumption at the anaerobic threshold (VO2 at AT) increased significantly for both groups. The low-intensity group saw an increase from 10 (IQR 9–11) to 12 (IQR 11–13), *p* = 0.004, and the high-intensity group saw an increase from 11 (IQR 10–12) to 13 (IQR 12–14), also with a *p*-value of 0.004. The respiratory exchange ratio (RER) saw significant reductions for both groups. The low-intensity group reduced from 1.20 (IQR 1.15–1.25) to 0.95 (IQR 0.90–1.00), *p* = 0.005, while the high-intensity group reduced from 1.30 (IQR 1.25–1.35) to 1.00 (IQR 0.95–1.05), also with a *p*-value of 0.005.

**Figure 3:**
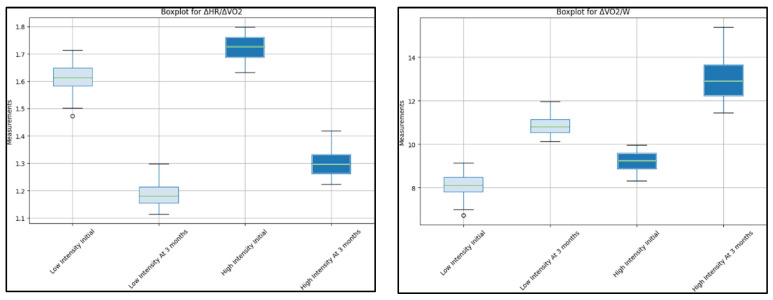
Comparison between initial cardiopulmonary testing and 3-month results between low-intensity and high-intensity rehabilitation groups.

Both groups showed a significant increase in heart rate (HR) from the maximum. The low-intensity group increased from 60 to 75 (*p* = 0.020), and the high-intensity group increased from 65 to 82 (*p* = 0.006). The ΔHR/ΔVO2 decreased significantly in both groups. The low-intensity group reduced from 1.60 (IQR 1.5–1.7) to 1.20 (IQR 1.1–1.3), *p* = 0.007, and the high-intensity group reduced from 1.7 (IQR 1.6–1.8) to 1.3 (IQR 1.2–1.4), *p* = 0.001. The ΔVO2/W (Figure 3) demonstrated significant increases for both groups. The low-intensity group improved from 8 (IQR 7–9) to 11 (IQR 10–12), *p* = 0.030, and the high-intensity group improved from 9 (IQR 8–10) to 14 (IQR 10–15), *p* = 0.008. Similarly, the ΔVO2/HR increased significantly for both groups. The low-intensity group improved from 9 (IQR 8–10) to 12 (IQR 11–13), *p* = 0.009, and the high-intensity group improved from 10 (IQR 9–11) to 13 (IQR 12–14), *p* = 0.045. Finally, the breathing reserve (BR) increased significantly in both groups. The low-intensity group improved from 15 (IQR 12–18) to 24 (IQR 22–26), *p*-value of less than 0.001, and the high-intensity group improved from 18 (IQR 15–21) to 29 (IQR 27–31), *p* = 0.010.

## 4. Discussion

### 4.1. Literature Findings

This study provided valuable insights into the characteristics of patients with severe COVID-19 and their recovery dynamics following cardiopulmonary rehabilitation. The observed improvements in lung function, inflammatory markers, and cardiopulmonary exercise testing parameters underscore the potential of structured rehabilitation programs in promoting recovery after severe COVID-19. Significant reductions in CRP and D-dimer levels post-rehabilitation were suggestive of diminished systemic inflammation and improved coagulation profiles. Furthermore, substantial improvements in pulmonary function measures and cardiopulmonary exercise testing parameters highlighted the beneficial effects of rehabilitation on cardiorespiratory fitness and endurance after 3 months. However, other studies have implemented shorter rehabilitation protocols. In a preliminary study involving almost 300 patients, the authors reported a 10% improvement in the six-minute walk distance (6MWD) during a 21-day rehabilitation program [23]. Similarly, another study found a substantial increase in the 6MWD of elderly patients referred for rehabilitation immediately after the acute phase of COVID-19 [24]. A longer rehabilitation program of six weeks showed further enhancement in the 6MWD and results of the pulmonary function tests, including Forced Vital Capacity (FVC) and Diffusing Capacity of the Lungs for Carbon Monoxide (DLCO) [25].

The stratification of the patients based on the intensity of the rehabilitation protocols unveiled similar outcomes between the high-intensity and low-intensity groups after three months of training. In general, the highest cardiac and pulmonary measurements were achieved after high-intensity training. However, despite the apparent superior improvements in terms of cardiopulmonary exercise testing variables, the data suggested that the low-intensity exercise regimen can expedite a quicker recovery and enhance the overall fitness in this patient population, for up to similar levels as in the high-intensity group if performed regularly for a minimum of three months. Notably, such lower-intensity interventions might be better tolerated by the study participants, reaffirming their safety and feasibility among patients recuperating from severe COVID-19.

The study’s findings substantiated the role of rehabilitation in addressing persistent dyspnea, a commonly reported post-acute sequelae in patients with severe COVID-19. By facilitating gradual restoration of pulmonary function and aerobic capacity, the implemented rehabilitation protocols potentially alleviated symptoms and enhanced the patients’ capacity for physical activity. This was further corroborated by the noticeable gains in the cardiopulmonary exercise testing parameters, which is indicative of the enhanced aerobic performance and respiratory efficiency. However, although our study insisted that patients undergo rehabilitation after hospital discharge in order to address dyspnea and other complications, other studies found that the timing (<30 days vs. ≥30 days) post-COVID-19 treatment before initiating rehabilitation did not affect the improved level [26]. This conclusion was based on increased measures of the 6MWD, vital capacity, and FEV1. Another study [27] confirmed that there was a significant improvement in the CRF level among patients with mild/moderate as well as severe/critical COVID-19 symptoms, again independent of when the rehabilitation began or the severity of the acute phase of the disease.

Despite the significant improvements reported in cardiac and pulmonary function, some issues remained that were reported in other studies. The normal range of these exploratory functions was not reached in all the measurements after three months. Similarly, other findings showed that men were more likely not to achieve a normal threshold of their respiratory function, as 25% of men vs. 13% of women in the analyzed population did not achieve the threshold [28]. Moreover, previous studies suggested that the mean 6MWD achieved at discharge was still below the predicted values [27]. Interestingly, the most pronounced improvement was noted in the patients with the most impaired CRF level and severe dyspnea at baseline, who followed cardiopulmonary rehabilitation models. However, the study by Spielmanns et al. [29] suggested that the patients with initially worse conditions achieved less frequent improvements in CRF levels despite undergoing intensive cardiac rehabilitation. This highlights the importance of implementing intensive cardiac rehabilitation, even later in the recovery period, considering the potential long-term impairment of CRF levels following severe respiratory infections [30,31].

Interestingly, no discernible differences were observed between vaccinated and unvaccinated individuals in the rehabilitation outcomes. Despite extensive data supporting the role of vaccination in reducing the severity of acute COVID-19 [32], its impact on the course of post-acute recovery remains relatively understudied. Importantly, the beneficial outcomes of rehabilitation were evident across varying age and gender groups, emphasizing the universality of these interventions. By customizing the rehabilitation protocols in line with the patients’ age-specific needs and potential comorbidities, we could ensure safety and efficacy across diverse demographic categories. Nevertheless, as a future perspective, the effectiveness of rehabilitation will be assessed as a function of clinical and functional improvement of the cardiopulmonary parameters, as well as assessing the potential protective factor of cardiopulmonary rehabilitation toward long-COVID syndrome.

### 4.2. Study Limitations

This study, despite its insightful findings, has certain limitations that need to be taken into account when interpreting the results. As a single-center study conducted in a specific hospital in Romania, the findings may not be applicable to the broader population of patients with severe COVID-19, especially those in different geographic locations or healthcare settings. The limited sample size, while sufficiently powered for the conducted analyses, may not provide precise estimates of the effect size or allow for stratified analyses based on varying comorbidities or demographic characteristics. The study’s selection criteria excluded patients with immunodeficiency diseases, those receiving immunosuppressive therapy, those with a history of chronic lung disease, and those with severe and unstable cardiovascular or respiratory conditions. Thus, the findings may not extend to these groups, who could also potentially benefit from cardiopulmonary rehabilitation.

The study followed up with patients only up to 3 months post-rehabilitation, offering no insights into the long-term effects of the interventions. Moreover, the focus was primarily on cardiopulmonary parameters, without including outcomes related to quality of life, mental health, and functionality. A notable limitation was the absence of a control group of patients not undergoing any rehabilitation, leaving the extent of the improvements that might occur naturally over time unclear. Potential bias might be introduced by the classification of COVID-19 severity based on clinical and paraclinical features, as some criteria could be subjective or influenced by patient cooperation or clinician interpretation. The statistical analyses, while rigorous, did not adjust for multiple comparisons, increasing the risk of a Type I error. Lastly, variability in the implementation of the standardized high-intensity and low-intensity rehabilitation protocols could influence the outcomes. These limitations should be factored in when interpreting the study’s findings and planning future research in this area.

## 5. Conclusions

Both low-intensity and high-intensity cardiopulmonary rehabilitation programs play a pivotal role in improving cardiopulmonary parameters in patients recovering from severe COVID-19. Although there was no significant difference between the two groups in some parameters, overall, the majority of the evaluated metrics showed an improvement after a 3-month period of rehabilitation. Parameters such as the peak oxygen consumption, respiratory exchange ratio, and breathing reserve demonstrated significant improvements. In addition, the markers of lung injury and oxygen need were significantly reduced in both groups, suggesting that such rehabilitation programs are conducive to mitigating the long-term pulmonary effects of severe COVID-19. Consequently, these findings underscore the critical importance of integrating structured, intensity-adjusted rehabilitation programs into the recovery plan for patients post severe COVID-19 to expedite their recuperation and enhance their quality of life.

## Figures and Tables

**Table 1:** Demographic and lifestyle variables.

Variables *	Low Intensity (*n* = 42)	High Intensity (*n* = 42)	*p*-Value
Age (mean ± SD)	56.3 ± 7.3	53.1 ± 10.8	0.115
Age (range)	42–67	35–72	-
Sex			0.659
Men (*n*, %)	23 (54.8%)	25 (59.5%)	
Women (*n*, %)	19 (45.2%)	17 (40.5%)	
BMI			0.669
Normal weight (18.5–24.9 kg/m^2^)	14 (33.3%)	12 (28.6%)	
Overweight (>24.9 kg/m^2^)	20 (47.6%)	24 (57.1%)	
Obese (>29.9 kg/m^2^)	8 (19.0%)	6 (14.3%)	
COVID-19 vaccination status			0.629
Vaccinated (2 doses)	11 (26.2%)	13 (31.0%)	
Unvaccinated	31 (73.8%)	29 (69.0%)	
Level of physical activity			0.512
Sedentary	26 (61.9%)	22 (52.4%)	
Active	12 (28.6%)	17 (40.5%)	
Very active	4 (9.5%)	3 (7.1%)	
Duration of hospitalization (mean ± SD)	11.6 ± 7.2	12.2 ± 6.8	0.695
Comorbidities (*n*, %)			0.708
Cardiovascular **	16 (38.1%)	13 (31.0%)	
Metabolic disease	10 (23.8%)	14 (33.3%)	
Digestive	6 (14.3%)	9 (21.4%)	
Others	8 (19.0%)	10 (23.8%)	
Antiviral medication requirement *			0.266
Yes	32 (76.2%)	36 (85.7%)	
No	10 (23.8%)	6 (14.3%)	

Data reported as *n* (%) and calculated using the Chi-square test and Fisher’s exact unless specified differently; BMI—Body Mass Index; SD—Standard Deviation; *—During the acute phase of COVID-19; **—Excluding unstable cardiac conditions.

**Table 2:** Laboratory data measured after SARS-CoV-2 viral clearance.

Variables	Normal Range	Low Intensity (*n* = 42)	Median (IQR)	High Intensity (*n* = 42)	Median (IQR)	*p*-Value
WBC (1000/mm^3^)	4.5–11.0	14 (33.3%)	11.4 (4.6)	16 (38.1%)	10.6 (4.8)	0.649
Lymphocytes (1000/mm^3^)	1.0–4.8	15 (35.7%)	6.1 (2.3)	11 (26.2%)	6.4 (2.5)	0.345
Hemoglobin (g/dL)	13.0–17.0	4 (9.5%)	14.1 (5.0)	3 (7.1%)	14.5 (5.2)	0.693
AST (U/L)	10–40	12 (28.6%)	33 (7)	13 (31.0%)	37 (8)	0.811
ALT (U/L)	7–35	13 (31.0%)	34 (10)	10 (23.8%)	36 (11)	0.463
Creatinine (µmol/L)	0.74–1.35	15 (35.7%)	1.09 (0.71)	18 (42.9%)	1.42 (0.76)	0.502
CRP (mg/dL)	0–10	18 (42.9%)	24 (16)	24 (57.1%)	28 (14)	0.190
IL-6 (pg/mL)	0.8–6.4	19 (45.2%)	9.8 (4.7)	21 (50.0%)	12.9 (5.8)	0.662
Procalcitonin (μg/L)	0–0.25	3 (7.1%)	0.10 (0.04)	5 (11.9%)	0.12 (0.06)	0.457
D-dimers (ng/mL)	<250	10 (23.8%)	231 (96)	12 (28.6%)	244 (101)	0.619
Ferritin (ng/mL)	20–250	14 (33.3%)	206 (48)	10 (23.8%)	225 (62)	0.334

Data reported as % outside the normal range and calculated using the Chi-square test and Fisher’s exact unless specified differently; IQR—Interquartile Range; WBC—White Blood Cells; AST—Aspartate aminotransferase; ALT—Alanine aminotransferase; CRP—C-reactive protein; IL—Interleukin.

**Table 3:** Initial pulmonary measurements of patients with severe COVID-19 at discharge.

Variables	Normal Range	Low Intensity (*n* = 42)	Median (IQR)	High Intensity (*n* = 42)	Median (IQR)	*p*-Value
Lung injury (%)	<5%	16 (38.1%)	21 (17–33)	18 (42.9%)	23 (16–32)	0.652
Oxygen need (%)	<21%	20 (47.6%)	30 (25–35)	22 (52.4%)	28 (23–33)	0.844
EF	>50%	10 (23.8%)	82 (78–86)	12 (28.6%)	84 (80–88)	0.670
SPAP	<25 mmHg	24 (57.1%)	30 (28–32)	26 (61.9%)	29 (27–31)	0.713
MVV	80–120 L/min	18 (42.9%)	75 (71–82)	20 (47.6%)	78 (73–83)	0.699
FVC	80–120% predicted	19 (45.2%)	73 (70–79)	21 (50.0%)	76 (72–81)	0.527
FEV1	80–120% predicted	20 (47.6%)	75 (70–80)	22 (52.4%)	74 (70–80)	0.735
FEV1/FVC ratio	70–80%	12 (28.6%)	68 (65–71)	14 (33.3%)	70 (67–73)	0.680
PEF	80–100% predicted	18 (42.9%)	76 (72–80)	20 (47.6%)	79 (75–83)	0.704
FEF 25–75	80–120% predicted	18 (42.9%)	78 (74–82)	20 (47.6%)	81 (77–85)	0.316

Data reported as % outside the normal range and calculated using the Chi-square test and Fisher’s exact unless specified differently; FE—Ejection fraction; SPAP—Systolic pulmonary artery pressure; MVV—Maximal voluntary ventilation; FVC—Forced vital capacity; FEV1—Forced expiratory volume in 1 s; PEF—Peak expiratory flow; FEF (25–75)—Forced expiratory flow at 25–75%.

**Table 4:** Pulmonary measurements of patients with severe COVID-19 after 3 months of rehabilitation.

Variables	Normal Range	Low Intensity (*n* = 42)	Median (IQR)	High Intensity (*n* = 42)	Median (IQR)	*p*-Value
Lung injury (%)	<5%	8 (19.0%)	14 (8–20)	6 (14.3%)	12 (10–17)	0.504
Oxygen need (%)	<21%	10 (23.8%)	20 (18–22)	8 (19.0%)	19 (17–21)	0.441
EF	>50%	4 (9.5%)	85 (82–88)	3 (7.1%)	87 (85–89)	0.496
SPAP	<25 mmHg	12 (28.6%)	25 (23–27)	10 (23.8%)	24 (22–26)	0.210
MVV	80–120 L/min	10 (23.8%)	85 (82–89)	8 (19.0%)	87 (84–89)	0.385
FVC	80–120% predicted	6 (14.3%)	85 (81–88)	7 (16.7%)	85 (80–88)	0.730
FEV1	80–120% predicted	10 (23.8%)	83 (82–86)	8 (19.0%)	87 (85–91)	0.708
FEV1/FVC ratio	70–80%	6 (14.3%)	75 (72–78)	5 (11.9%)	76 (74–78)	0.662
PEF	80–100% predicted	8 (19.0%)	81 (78–85)	6 (14.3%)	81 (79–85)	0.317
FEF 25–75	80–120% predicted	5 (11.9%)	84 (82–90)	6 (14.3%)	83 (81–87)	0.301

Data reported as % outside the normal range and calculated using the Chi-square test and Fisher’s exact unless specified differently; FE—Ejection fraction; SPAP—Systolic pulmonary artery pressure; MVV—Maximal voluntary ventilation; FVC—Forced vital capacity; FEV1—Forced expiratory volume in 1 s; PEF—Peak expiratory flow; FEF (25–75)—Forced expiratory flow at 25–75%.

**Table 5:** Initial cardiopulmonary exercise testing results of patients with severe COVID-19 at discharge.

Variables	Normal Range	Low Intensity (*n* = 42)	Median (IQR)	High Intensity (*n* = 42)	Median (IQR)	*p*-Value
Work intensity (W)	10–60	20 (47.6%)	27 (22–29)	22 (52.4%)	43 (41–46)	<0.001
VO2 (peak)	>20 mL/kg/min	22 (52.4%)	18 (17–19)	24 (57.1%)	19 (18–20)	0.294
VO2 (from predicted)	80–120%	24 (57.1%)	75 (70–80)	26 (61.9%)	78 (73–83)	0.708
VO2 at AT	>11 mL/kg/min	20 (47.6%)	10 (9–11)	22 (52.4%)	11 (10–12)	0.460
RER	0.8–1.15	16 (38.1%)	1.2 (1.15–1.25)	18 (42.9%)	1.3 (1.25–1.35)	0.692
HR (from max.)	60–90%	20 (47.6%)	60 (55–65)	22 (52.4%)	65 (60–70)	0.347
ΔHR/ΔVO2	1–1.5	18 (42.9%)	1.6 (1.5–1.7)	21 (50.0%)	1.7 (1.6–1.8)	0.422
ΔVO2/W	10–15 mL/min/W	20 (47.6%)	8 (7–9)	19 (45.2%)	9 (8–10)	0.783
ΔVO2/HR	10–15 mL/beat	20 (47.6%)	9 (8–10)	22 (52.4%)	10 (9–11)	0.419
BR	20–40%	22 (52.4%)	15 (12–18)	24 (57.1%)	18 (15–21)	0.301

Data reported as % outside the normal range and calculated using the Chi-square test and Fisher’s exact unless specified differently; W—Watts; VO2 (peak)—Oxygen consumption at peak exercise; VO2 at AT—Oxygen consumption at the anaerobic threshold; RER—Respiratory exchange ratio; HR—Heart rate; BR—Breathing reserve.

**Table 6:** Final cardiopulmonary exercise testing results of patients with severe COVID-19 after 3 months of rehabilitation.

Variables	Normal Range	Low Intensity (*n* = 42)	Median (IQR)	High Intensity (*n* = 42)	Median (IQR)	*p*-Value
Work intensity (W)	10–60	15 (35.7%)	38 (36–44)	12 (28.6%)	47 (44–51)	0.094
VO2 (peak)	>20 mL/kg/min	12 (28.6%)	21 (20–22)	10 (23.8%)	23 (22–24)	0.331
VO2 (from predicted)	80–120%	13 (31.0%)	85 (80–90)	11 (26.2%)	90 (85–95)	0.416
VO2 at AT	>11 mL/kg/min	9 (21.4%)	12 (11–13)	7 (16.7%)	13 (12–14)	0.208
RER	0.8–1.15	14 (33.3%)	0.95 (0.90–1.00)	12 (28.6%)	1.00 (0.95–1.05)	0.190
HR (% from max.)	60–90%	16 (38.1%)	75 (70–80)	13 (31.0%)	82 (78–86)	0.106
ΔHR/ΔVO2	1–1.5	11 (26.2%)	1.2 (1.1–1.3)	9 (21.4%)	1.3 (1.2–1.4)	0.325
ΔVO2/W	10–15 mL/min/W	12 (28.6%)	11 (10–12)	10 (23.8%)	14 (10–15)	0.282
ΔVO2/HR	10–15 mL/beat	14 (33.3%)	12 (11–13)	11 (26.2%)	13 (12–14)	0.440
BR	20–40%	13 (31.0%)	24 (22–26)	10 (23.8%)	29 (27–31)	0.273

Data reported as % outside the normal range and calculated using the Chi-square test and Fisher’s exact unless specified differently; W—Watts; VO2 (peak)—Oxygen consumption at peak exercise; VO2 at AT—Oxygen consumption at the anaerobic threshold; RER—Respiratory exchange ratio; HR—Heart rate; BR—Breathing reserve.

**Table 7:** Comparison between initial cardiopulmonary measurements and 3-month results between low-intensity and high-intensity rehabilitation groups.

Variables	Low Intensity Initial (*n* = 42)	Low Intensity at 3 Months (*n* = 42)	*p*-Value	High Intensity Initial (*n* = 42)	High Intensity at 3 Months (*n* = 42)	*p*-Value
Lung injury (%)	21 (17–33)	14 (8–20)	0.005	23 (16–32)	12 (10–17)	0.001
Oxygen need (%)	30 (25–35)	20 (18–22)	0.002	28 (23–33)	19 (17–21)	0.003
FE	82 (78–86)	85 (82–88)	0.010	84 (80–88)	87 (85–89)	0.020
SPAP	30 (28–32)	25 (23–27)	0.015	29 (27–31)	24 (22–26)	0.011
MVV	75 (71–82)	85 (82–89)	<0.001	78 (73–83)	87 (84–89)	0.008
FVC	73 (70–79)	85 (81–88)	0.004	76 (72–81)	85 (80–88)	0.007
FEV1	75 (70–80)	83 (82–86)	0.009	74 (70–80)	87 (85–91)	<0.001
FEV1/FVC ratio	68 (65–71)	75 (72–78)	<0.001	70 (67–73)	76 (74–78)	0.010
PEF	76 (72–80)	81 (78–85)	0.012	79 (75–83)	81 (79–85)	0.020
FEF 25–75	78 (74–82)	84 (82–90)	0.005	81 (77–85)	83 (81–87)	0.022

Data reported as median (IQR) and calculated using Mann–Whitney u-test; FE—Fraction of expired oxygen; SPAP—Systolic pulmonary artery pressure; MVV—Maximal voluntary ventilation; FVC—Forced vital capacity; FEV1—Forced expiratory volume in 1 s; PEF—Peak expiratory flow; FEF (25–75)—Forced expiratory flow at 25–75%.

**Table 8:** Comparison between initial cardiopulmonary testing and 3-month results between low-intensity and high-intensity rehabilitation groups.

Variables	Low Intensity Initial (*n* = 42)	Low Intensity at 3 Months (*n* = 42)	*p*-Value	High Intensity Initial (*n* = 42)	High Intensity at 3 Months (*n* = 42)	*p*-Value
Work intensity (W)	27 (22–29)	38 (36–44)	<0.001	43 (41–46)	47 (44–51)	0.135
VO2 (peak)	18 (17–19)	21 (20–22)	0.010	19 (18–20)	23 (22–24)	0.002
VO2 (from predicted)	75 (70–80)	85 (81–90)	0.003	78 (73–83)	90 (85–95)	<0.001
VO2 at AT	10 (9–11)	12 (11–13)	0.004	11 (10–12)	13 (12–14)	0.004
RER	1.20 (1.15–1.25)	0.95 (0.90–1.00)	0.005	1.3 (1.25–1.35)	1.00 (0.95–1.05)	0.005
HR (% from max.)	60 (55–65)	75 (70–80)	0.020	65 (60–70)	82 (78–86)	0.006
ΔHR/ΔVO2	1.6 (1.5–1.7)	1.2 (1.1–1.3)	0.007	1.7 (1.6–1.8)	1.3 (1.2–1.4)	0.001
ΔVO2/W	8 (7–9)	11 (10–12)	0.030	9 (8–10)	14 (10–15)	0.008
ΔVO2/HR	9 (8–10)	12 (11–13)	0.009	10 (9–11)	13 (12–14)	0.045
BR	15 (12–18)	24 (22–26)	<0.001	18 (15–21)	29 (27–31)	0.010

Data reported as median (IQR) and calculated using Mann–Whitney u-test; W—Watts; VO2 (peak)—Oxygen consumption at peak exercise; VO2 at AT—Oxygen consumption at anaerobic threshold; RER—Respiratory exchange ratio; HR—Heart rate; BR—Breathing reserve.

## Data Availability

Data available on request.
